# Parched Corn Meal

**Published:** 1863-05

**Authors:** 


Parched Corn Meal.—Any one who has traveled over the western prairies is undoubtedly familiar with the article of food named at the head of this article. The mode of preparing it is to parch the corn, generally in an ash bath, reduce it to meal, and add a due proportion of sugar. Provided with this simple article of diet the Indians, hunters and trappers of the West will travel hundreds of miles, a very small quantity in bulk sufficing for many days. It is, withal, exceedingly palatable, and is usually mixed in water when eaten.

This would be an excellent addition to the rations of our soldiers, taking the place of both flour and coffee. A small quantity of it will go a great ways, and its use would economize money, time, bulk, and weight, all considerations of importance, the three latter, especially so in rapid army movements.—*Medical and Surgical Reporter.*

